# Molecular analysis of clinical *Burkholderia pseudomallei* isolates from southwestern coastal region of India, using multi-locus sequence typing

**DOI:** 10.1371/journal.pntd.0006915

**Published:** 2018-11-12

**Authors:** Aayushi Kamthan, Tushar Shaw, Chiranjay Mukhopadhyay, Subodh Kumar

**Affiliations:** 1 Microbiology Division, Defence Research & Development Establishment, Gwalior, Madhya Pradesh, India; 2 Department of Microbiology, Kasturba Medical College, Manipal Academy of Higher Education, Manipal, India; University of Texas Medical Branch, UNITED STATES

## Abstract

**Background:**

The Gram-negative soil dwelling bacterium *Burkholderia pseudomallei* is the etiological agent of melioidosis. The disease is endemic in most parts of Southeast Asia and northern Australia. Over last few years, there has been an increase in number of melioidosis cases from India; however the disease epidemiology is less clearly understood. Multi-locus sequence typing (MLST) is a powerful genotypic method used to characterize the genetic diversity of *B*. *Pseudomallei* both within and across the geographic regions.

**Methods:**

In this study, MLST analysis was performed on 64 *B*. *pseudomallei* clinical isolates. These isolates were obtained between 2008–2014 from southwestern coastal region of India. Broad population patterns of Indian *B*. *pseudomallei* isolates in context with isolates of Southeast Asia or global collection was determined using *in silico* phylogenetic tools.

**Results:**

A total of 32 Sequence types (STs) were reported among these isolates of which 17 STs (53%) were found to be novel. ST1368 was found as group founder and the most predominant genotype (n = 11, 17%). Most of the *B*. *pseudomallei* isolates reported in this study (or other Indian isolates available in MLST database) clustered in one major group suggesting clonality in Indian isolates; however, there were a few outliers. When analyzed by measure of genetic differentiation (F_ST_) and other phylogenetic tools (e.g. PHYLOViZ), Indian STs were found closer to Southeast Asian isolates than Australian isolates. The phylogenetic analysis further revealed that within Asian clade, Indian isolates grouped more closely with isolates from Sri Lanka, Vietnam, Bangladesh and Thailand.

**Conclusions:**

Overall, the results of this study suggest that the Indian *B*. *pseudomallei* isolates are closely related with lesser heterogeneity among them and cluster in one major group suggesting clonality of the isolates. However, it appears that there are a few outliers which are distantly related to the majority of Indian STs. Phylogenetic analysis suggest that Indian isolates are closely related to isolates from Southeast Asia, particularly from South Asia.

## Author summary

*Burkholderia pseudomallei*, a gram negative bacterium, is the causative agent of melioidosis. *B*. *pseudomallei* is a soil saprophyte and causes infections in humans by percutaneous inoculation, inhalation or ingestion. Melioidosis is a life threatening disease, which requires prolonged antibiotic treatment and is classically characterized by pneumonia, septicemia and multiple abscesses. Melioidosis is widely prevalent in Southeast Asia and northern Australia. Of late it has been reported from tropical, subtropical and temperate regions. The predicted annual global burden of melioidosis is 165,000 cases. *B*. *pseudomallei* has been classified as a Category B threat agent by US Center for Disease Control. Melioidosis is an emerging disease in India that affects many regions. Over the past few years, there has been an increase in number of melioidosis cases, mainly from southwestern costal part of India. This study provides new insights into molecular epidemiology of melioidosis in India. By use of multi locus sequence typing (MLST), we show that Indian isolates are closely related and cluster in one major group suggesting clonality of the isolates. We further show that Indian isolates are more closely related to isolates from Asian countries particularly from South Asia.

## Introduction

Melioidosis, caused by soil saprophyte Gram-negative bacterium *Burkholderia pseudomallei*, is classically characterized by pneumonia, septicemia and multiple abscesses. There are various predisposing factors for melioidosis with diabetes mellitus as one of the most important factors. Until recently, the disease was considered endemic only to Southeast Asia and northern Australia, however now it has been reported from tropical, subtropical and temperate regions [1,2]. Reports of melioidosis from India had been few and sporadic [3,4], however, over the past few years, there has been an increase in number of melioidosis cases. It has been reported from various states of India including Karnataka, Kerala, Maharashtra, Tamil Nadu and Puducherry [5]. The disease is quite prevalent in southwestern costal part of the country and is strongly associated with rainfall and diabetes mellitus [5,6]. A mathematical modeling study of 2015 has predicted global annual burden of melioidosis to be 165,000 cases with 89,000 deaths with Indian subcontinent to have highest burden of the disease [7].

Regional variations in melioidosis signs and symptoms have been reported. It has been seen that while prostatic abscess and encephalomyelitis are common in Australians whereas parotid abscess and hepatosplenic suppuration are most frequently seen in patients from Thailand [8]. The reasons for this diversity remain unclear but it could be due to host, bacterial and environmental factors [9]. Study of epidemiology by molecular methods provides insight about bacterial diversity and distribution. Various molecular methods e.g. pulse field electrophoresis, ribotyping have been used for phylogenetic reconstruction with different levels of success, however, multi-locus sequence typing (MLST) is a proven tool for the molecular typing of *B*. *pseudomallei*. MLST not only helps to explore the sequence types (STs) in a population or helps tracing an outbreak [10] but also helps to understand the microbial evolution. There is large MLST database for *B*. *pseudomallei* (http://pubmlst.org/bpseudomallei/).

In India, melioidosis is an emerging endemic infection and potentially fatal as early diagnosis is missed due to its varied manifestations such as localized or disseminated infection. Here, it has largely been reported from the coastal regions and it is generally believed that the disease is underreported or misdiagnosed. Using MLST, we had previously reported in a pilot study that Indian isolates were genetically diverse from the Australian or Southeast Asian isolates [11]. In this work, we aimed to study the genetic diversity among larger number of *B*. *pseudomallei* isolates using MLST. Further, using sequence data we attempted to find broad population patterns of Indian isolates with global collection of 5541 isolates and construct phylogeny of *B*. *pseudomallei* Indian isolates and their close relatives from Southeast Asia.

## Methods

### Ethics statement

This study was approved by Institutional Ethical Committee of Kasturba Hospital, Manipal under protocol number IEC 141/2011.

### Bacterial strains and growth conditions

Sixty-four (n = 64) clinical isolates of *B*. *pseudomallei* isolated from Karnataka and adjacent states (Kerala, Goa and Puducherry) of southern India were included in this study. These isolates were collected from melioidosis patients at Kasturba Medical College, Manipal, Karnataka during 2008–2014. All the isolates were identified using API20NE and later confirmed using TTS1-PCR assay [12] and latex agglutination test from the colonies. The isolates were also characterized for genetic markers linked to geographic origin *Yersinia-*like fimbriae (YLF) and *B*. *thailendensis-*like flagellum and chemotaxis (BTFC) gene [13].

For preparation of DNA, bacteria were grown overnight in Luria- Bertani (LB) broth at 37°C in high containment facility, a biosafety level 3 facility at Defence Research & Development Establishment (DRDE), Gwalior. Genomic DNA was isolated from culture using DNeasy blood and tissue genomic DNA kit (Qiagen Gmbh, Hilden), according to the manufacturer’s instructions. The genomic DNAs were stored at -20°C till further used.

### Multi locus sequence typing (MLST)

MLST was carried out according to the method of Godoy *et al* [14]. Primer sequences targeting the conserved regions of seven housekeeping genes *(ace- gltB- gmhD- lepA- lipA- narK- ndh*) of *B*. *pseudomallei* were used as shown in MLST site (http://pubmlst.org/bpseudomallei/). PCR amplification, sequence analysis and determination of ST for each isolate were carried out by our earlier reported procedure [11]. The purified PCR amplicons were double pass sequenced using commercial SANGER sequencing services (M/s Genotypic Technology Pvt. Ltd., Bengaluru).

### Phylogenetic analysis

The relatedness of MLST profiles of isolates of this study or Indian isolates in *B*. *pseudomallei* MLST database was performed using eBURST software [15] (launched at http://eburst.mlst.net/) with single locus variant (SLV) selected. eBURST or global optimal based upon related sequence type (goeBURST) allows for an unrooted tree-based representation of the relationship of the analyzed isolates. The diversification of the "founding" genotype is reflected in the appearance of STs differing only in one housekeeping gene sequence from the founder genotype–single locus variants (SLVs). Further diversification of those SLVs results in the appearance of variations of the original genotype with more than one difference in the allelic profile: double locus variants (DLVs), triple locus variants (TLVs) and so on. The final eBURST tree provides a hypothetical pattern of descent for the strains analyzed. Basic quantities such as number of alleles, number of variable sites per allele, number and frequency of single nucleotide polymorphism (SNPs) in each locus were determined. Nucleotide sequence diversity (π) was calculated using DNAsp V5.1 software [16]. The association of individual STs with the type of infection among the study population was carried out using Fisher exact test (Graph Pad Prism software, La Jolla, USA).

Measures of genetic differentiation (F_ST_) between the concatenated sequences of Indian origin and from Asia or Australia was estimated using DNAsp V5.1 [16]. Relationship of Indian STs with global collection of STs was studied using goeBURST [17] implemented in PHYLOViZ programme [18] available at MLST site (http://pubmlst.org/bpseudomallei). PHYLOViZ is a flexible and expandable plugin based tool that is able to handle large datasets and builds on goeBURST implementation. In order to further determine the relationship of Indian STs with STs from Asia and Australia, topology and grouping of all Indian STs were displayed on constructed boot strapped phylogenetic tree using Unweighted Pair Group Method with Arithmetic average (UPGMA) method in molecular evolutionary genetic analysis version -6 (MEGA 6) software [19]. Indian STs including STs of this study were analyzed with selected 57 STs from other Asian countries e.g. Sri Lanka, Vietnam, Bangladesh, Cambodia, Malaysia, Thailand, China and Laos and, 6 most predominant STs from Australia. The 57 STs were either SLV or DLV of the Indian STs. Concatenated sequences of all STs used for this study were downloaded from MLST site (http://pubmlst.org/bpseudomallei/).

## Results

The present study involved MLST analysis of 64 clinical isolates of *B*. *pseudomallei* obtained during the time span of seven years (2008–2014) from India. These isolates were from states of Karnataka (84%), Kerala (6.25%), Goa (6.25%) and Puducherry (1.5%). Among the 64 isolates, 31 (48.4%) were obtained from patients with bacteremic melioidosis and 27 (42.2%) from patients with localized form of melioidosis. All *B*. *pseudomallei* isolates belonged to the Asian biogeographic YLF variant. Isolate study based on the clinical condition, geographical location, year of isolation, YLF/BTFC genotype and sequence type are enlisted in S1 Table.

Among obtained STs,—the numbers of alleles per locus varied from 2 to 4 and SNPs ranged from 1 to 7. The level of locus sequence diversity for each of the 7 loci was found to be around 2% (Table 1). Nucleotide diversity (π) among Indian isolates of this study was found to be 0.00137 and changed to 0.00329 for total Indian isolates in the database.

**Table 1:** Properties of the MLST loci in the clinical *B*. *pseudomallei* isolates of this study.

Locus	No. of nucleotide analyzed	No of Alleles	No. of SNP	SNP Frequency^a^	No. of variable sites	Sequence diversity rate^b^
*ace*	519	2	1	0.19%	1	1.96%
*gltB*	522	4	5	0.95%	3	2.32%
*gmhD*	468	3	7	1.49%	3	2.17%
*lepA*	486	3	3	0.61%	2	2.47%
*lipA*	402	4	5	1.24%	4	2.22%
*narK*	561	3	3	0.53%	2	2.23%
*ndh*	443	2	1	0.22%	1	2.24%

^a^Rate of SNPs diversity in relation with locus length

^b^Rate of allele diversity in relation with the number of total referenced database alleles

### MLST analysis

Among 64 isolates of this study, a total of 32 STs were identified. The frequencies of STs among isolates ranged from 1–11 with ST1368 (n = 11), ST42, ST1373, ST1478 (n = 4), ST124, ST1512, ST1507, ST1375 (n = 3) being the most predominant STs. Seventeen STs identified in this study (ST1373, ST1374, ST1506, ST1507, ST1508, ST1509, ST1510, ST1511, ST1512, ST1513, ST1514, ST1515, ST1516, ST1517, ST1518, ST1519, ST1520) were found to be novel that were not reported previously and 15 STs were previously documented, five of which were reported as novel in our previous study [11].

### Genetic relatedness among STs from India

**Fig 1:**
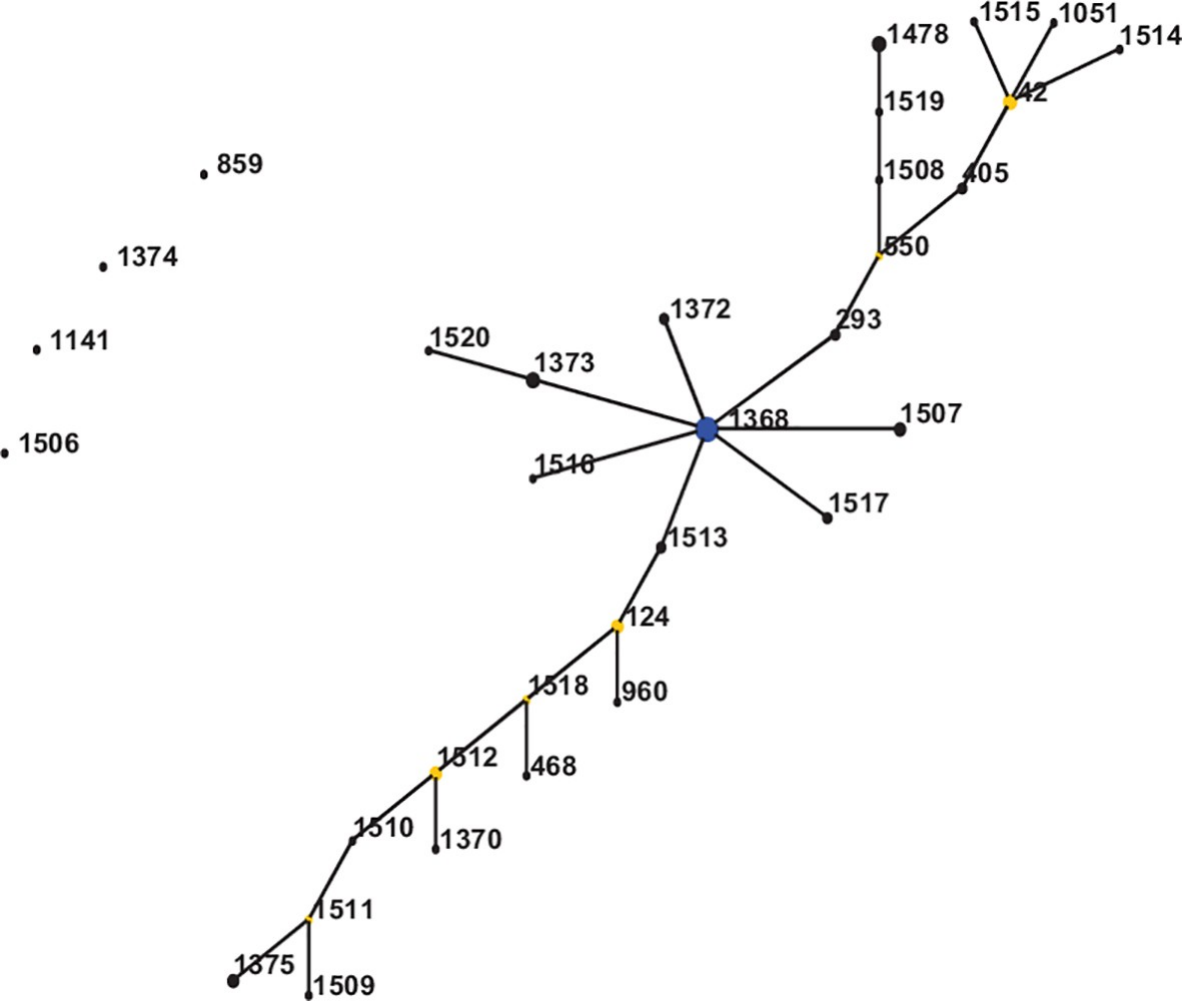
Genetic relationship of *B*. *pseudomallei* isolates of this study using eBURST. Blue dot refers to group founder and yellow dot refers to sub-group founder. Each black dot represents single genotype.

When analyzed by eBURST, almost all STs of this study formed in one large group with ST1368 as predicted founder; only ST1141, ST1374, ST1506 and ST859 were found to be singleton. ST1368 had 7 SLV, 4 DLV, 7 TLV and 9 satellites STs. ST550, ST124, ST42, ST1511, ST1512, and ST1518 were found to be subgroup founders. Interestingly, all novel 17 STs of this study except ST1506 and ST1374 also clustered in the same group (Fig 1, S2 Table).

**Fig 2:**
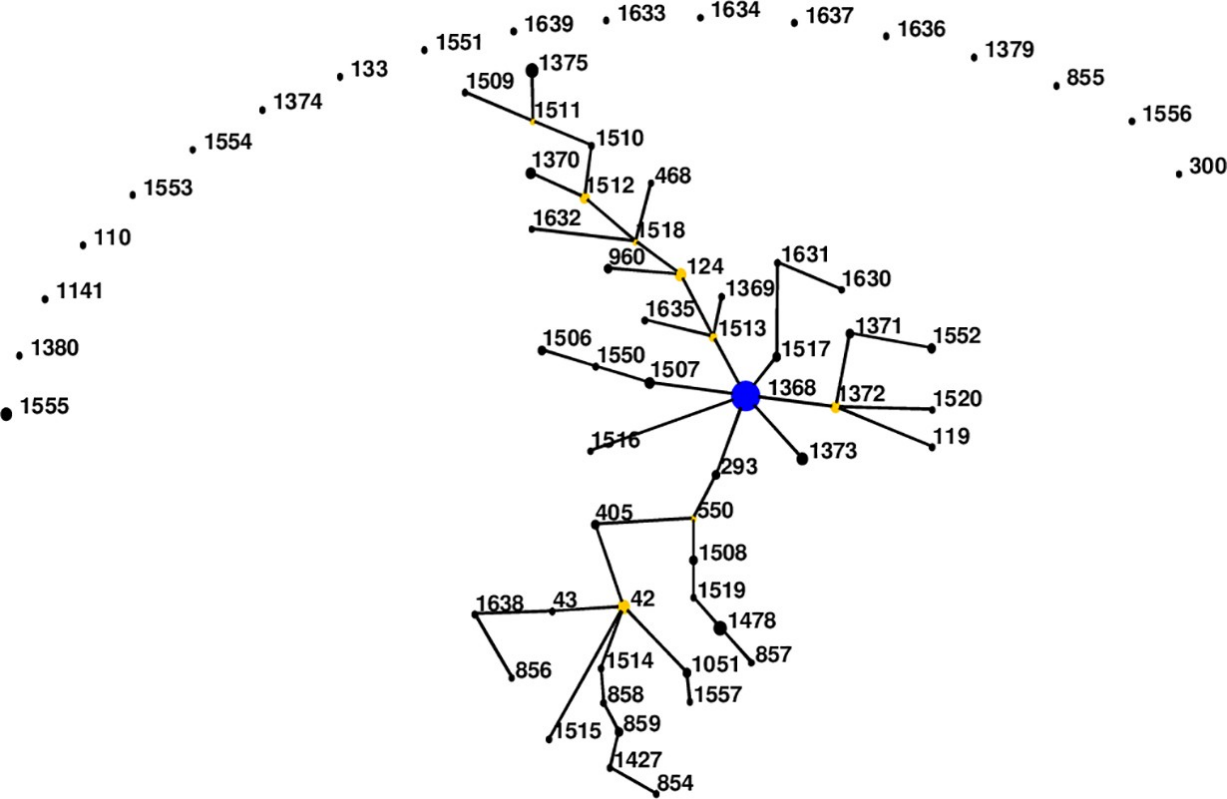
Genetic relationship of all Indian *B*. *pseudomallei* isolates (n = 130) using eBURST. Blue dot refers to group founder and yellow dot refers to sub-group founder. Each black dot represents single genotype. Re-sampling for bootstrapping = 10,000; minimum number of identical loci for group definition = 6; minimum number of SLV for subgroup definition = 3.

A total of 130 isolates are now available (as on 16^th^ October, 2018) in *B*. *pseudomallei* MLST database from India which include data from 64 isolates of this study. All isolates from India except one, which was isolated from soil, were clinical isolates. Of the 65 STs, 47 STs (109 isolates) cluster in one group with the remaining 18 STs (21 isolates) being singleton. ST1368 is dispersed all over the four Indian states of Karnataka, Kerala, Goa and Puducherry (S1 Table), [11]. It had a frequency of 26 with 7 SLV, 10 DLV, 11 TLV and 18 satellites STs. The present study has expanded the clonal cluster of Indian isolates by adding more branching STs. eBURST analysis of total Indian isolates revealed ST1513 and ST1372 to be as additional sub-group founders (Fig 2).

### Genetic relatedness of Indian STs with global collection

**Fig 3:**
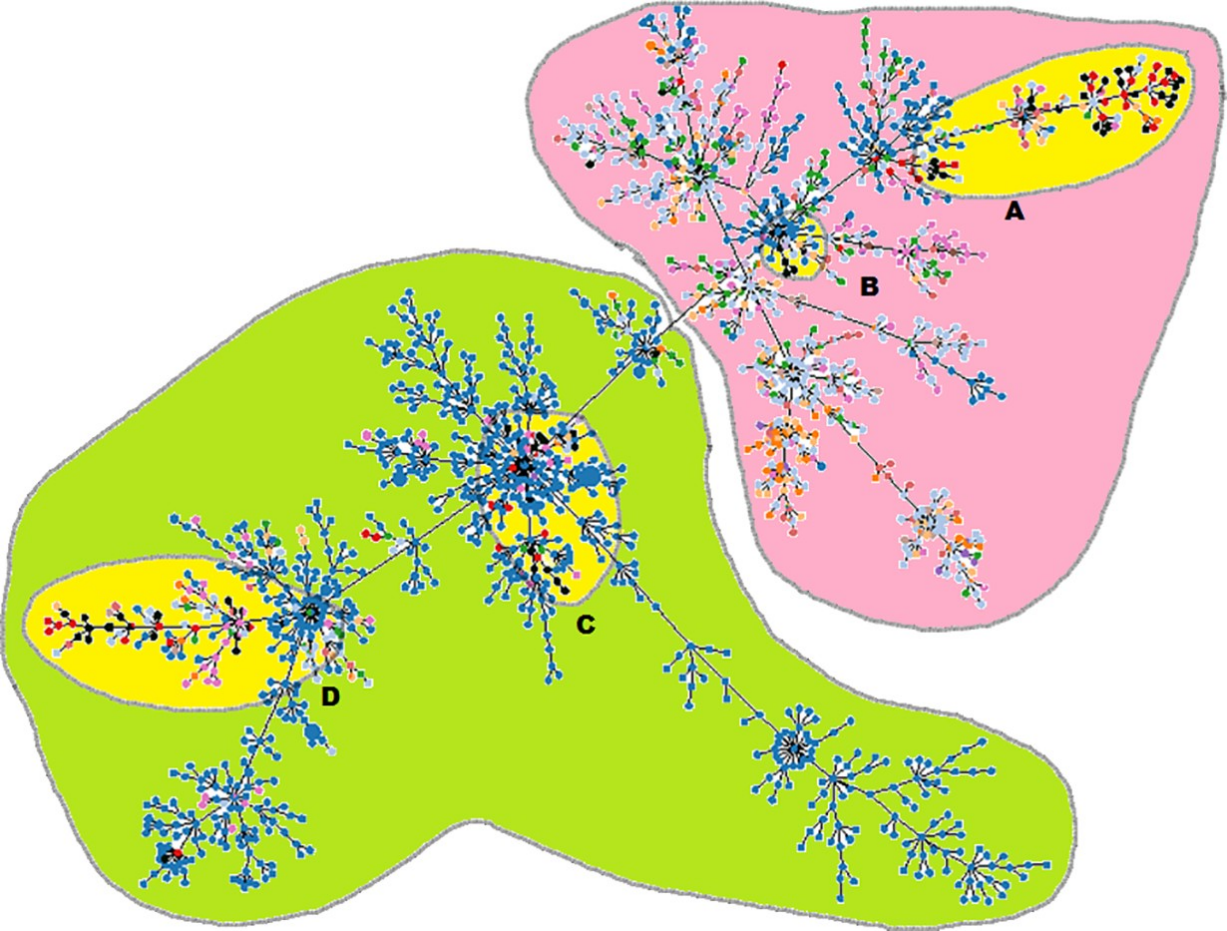
PHYLOViZ analysis showing the genetic relationship among global collection of sequence types (STs) of *Burkholderia pseudomallei*. Each dot represents a distinct ST. Oceania and Southeast Asian dominant STs are shaded in green and pink, respectively. Indian STs (shaded in yellow) cluster in four groups- Groups A & B cluster with STs from Southeast Asia and Groups C & D cluster with STs from Australia. STs from India are colored in black. Different colored dots represent STs from Sri Lanka (red), Australia (blue), Thailand (light blue), Malaysia (orange), China (light orange), Cambodia (green), Vietnam (light mehroon), Singapore (purple), Bangladesh (light purple), Laos (mehroon) and other countries (light pink).

Measure of genetic differentiation (F_ST_) between Indian and Australian or Asian isolates was determined and was found to be 0.1561 and 0.09082 respectively, suggesting that Indian isolates are closer to Asian clade rather than Australian clade. When Indian STs were analyzed by PHYLOViZ with the global collection of 5541 isolates in the *B*. *pseudomallei* MLST isolates database (as on 19^th^ May, 2018), majority of Indian isolates grouped in four groups (Fig 3). Group A (ST42, ST550, ST1051, ST1370, ST1555) and B (ST405, ST856, ST1637) clustered with Asian clade and accounted for about 43% (28/65) of the known STs. Indian isolates appeared to be different from the main Thailand cluster and grouped closure to isolates from South Asia e.g. Sri Lanka, Bangladesh. There was overlap of Indian STs with STs of other Asian countries e.g. Bangladesh (ST42, ST43, ST300); Vietnam (ST550, ST858, ST1051); Sri Lanka (ST293, ST1141); Thailand (ST300, ST405) and China (ST405).

About 57% of Indian STs grouped with Australian STs in two groups (Group C–ST124, ST1375, ST1507, ST1512, ST1552, ST1554 and Group D—ST1368, ST1372, ST1373). ST468 and ST1051 were the only Indian STs that overlapped with Australia. However, all isolates of this study were YLF positive, a gene cluster found predominantly among isolates of Southeast Asian origin. Some of the Indian STs also had overlapping STs with USA; these included ST1427, ST550 and ST960. *B*. *pseudomallei* has never been found in the US environment, thus all of these USA patient melioidosis infections would have been acquired overseas.

We also wanted to find out whether predominant Indian STs had SLVs with STs of other countries. SLV of ST1368, ST1373 (ST293), ST1478 (ST1147, ST1137), ST1507 (ST1138, ST1152) and ST1512 (ST1152) were reported from Sri Lanka. A few Indian STs had SLVs with STs from Thailand (ST42-ST405, ST501, ST371), Bangladesh (ST42-ST43) and Vietnam (ST1373- ST1568). Only a few predominant STs of other countries had SLVs with Indian STs which included STs from Sri Lanka (ST1132-ST1514, ST1517, ST1630), Thailand (ST371-ST1514) and Vietnam (ST41- ST1051).

**Fig 4:**
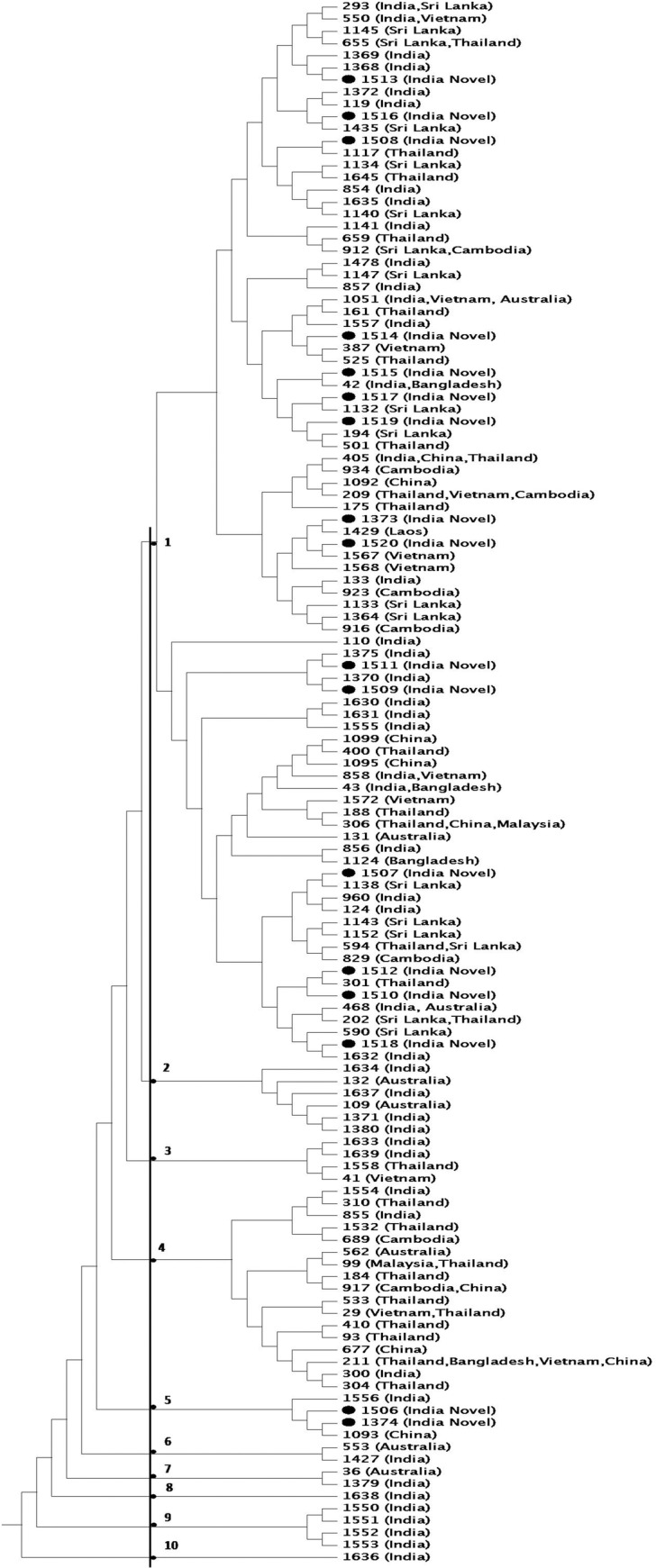
The evolutionary history inferred using the UPGMA method. The analysis involved 128 nucleotide sequences of 65 Indian STs and 63 STs from Sri Lanka, Vietnam, Bangladesh, Cambodia, Malaysia, Thailand, China, Laos and Australia.

The topology and grouping of all STs from India was displayed with selected STs from Asia and Australia and results are shown in Fig 4. This type of cluster analysis presents several advantages, such as the ease of interpretation and the creation of an hierarchical grouping of the isolates that can provide a global overview of the relatedness of the isolates under study and how the defined clusters are connected to each other. It was found that majority of the Indian isolates grouped in ‘Group 1’along with STs from Sri Lanka, Vietnam, Bangladesh, Cambodia, Malaysia, Thailand, China and Laos. ‘Group 4’ was another group in which STs from India and other Asian countries clustered together. Both of these groups had one ST each from Australia. ‘Groups 2, 6 & 7’ which were relatively smaller groups, had STs only from India and Australia. ‘Groups 8, 9 & 10’ had STs only from India, which probably represent some distantly related STs from main Indian cluster.

### *B*. *pseudomallei* STs and disease association

Attempts were also made to find association between the STs and the clinical conditions. In this study, ST1368 was found to be predominantly associated with localized infections (8 of 27), however this association was not statistically significant (P = 0.913). We also did not observe any association of STs with geographical location and year of isolation.

## Discussion

MLST is an unambiguous and powerful procedure to study the bacterial populations and global epidemiology [14]. It has been extensively utilized to characterize *B*. *pseudomallei* and analysis of MLST data by different phylogenetic tools such as eBURST, goeBURST and PHYLOViZ has succeeded in making the genetic relatedness of *B*. *pseudomallei* from distinct geographical locations. The present study was undertaken to characterize 64 clinical isolates of *B*. *pseudomallei* by MLST from various geographical locations in the southwestern coastal region of India. Here, we wanted to understand the diversity of genotypes of India and their relatedness to STs from Asia or Australia.

In this study, 32 STs were identified and overall diversity of isolates was 0.5 STs/isolate. The low level of locus sequence diversity and nucleotide diversity (π) for STs indicates low diversity among Indian isolates. When analysed by eBURST, an algorithm which provides a hypothetical pattern of descent for the analyzed strains, most of the isolates of this study (or all Indian isolates available in MLST database) formed one clonal complex suggesting limited genotypic diversity. One of a predominant ST (ST1630) reported as singleton in a recent study [20], which was isolated from southern states of Tamil Nadu and Andhra Pradesh, also appeared to be part of this clonal complex. Only 18 Indian STs were found to be singleton and were outliers. These singleton STs also included ST300 which was reported to be group founder in above study [20]. Topology and grouping of all Indian STs with other Asian and Australian STs by MEGA showed that majority of Indian isolates grouped together in one large group, however few STs were distant and unique particularly ST1636. ST1368 was found to be the predominant ST representing about 17% of the total isolates. Predominance of particular genotype among single population communities have previously been reported from various endemic areas of Malaysia [21], Sri Lanka [22], Thailand, and Australia (http://pubmlst.org/bpseudomallei/).Occurrence of ST1368 over a period of time, which appeared from an isolate of 2006 [11] and in all 4 states of India suggests high level of genetic uniformity among *B*. *pseudomallei* isolates from southwestern region of India.The results of this study suggest that the Indian isolates are closely related with lesser heterogeneity among them and belong to single group. However, it appears that there are a few outliers which are distantly related to the majority of Indian STs.

Attempts were made to find out the association between common STs (e.g. ST1368, ST42, ST124) of this study and disease presentation, however like our previous work [11], no such association could be observed. One of the reasons for this lack of association could be small sample size. Preliminary results of this study could only be strengthened if larger STs data is available and correlated with disease presentation. Except a few studies [22], most studies, however, could not find any association between STs and disease presentation.

Measure of genetic differentiation (F_ST_), presence of YLF genetic marker in *B*. *pseudomallei* isolates, PHYLOVIZ analysis and phylogenetic clustering by MEGA suggested Indian isolates to be closer to Asian clade rather than Australian clade. PHYLOViZ analysis further revealed that within Asian clade, Indian isolates grouped were more closely with isolates from Sri Lanka, Vietnam, Bangladesh and Thailand. There were many STs which overlapped with these countries. Some of the overlapping STs (ST300, ST405) were first isolated in Thailand in 1965, while other STs (e.g. ST42, ST293, ST550) were isolated in Bangladesh, Sri Lanka and Vietnam only after late 1990s suggesting continued migration of bacteria among India and Southeast Asian countries. These results are however, contrary to our previous findings[11], which suggested Indian strains to be distinct from Southeast Asian or Australasian isolates. This may be due to better analysis of available (and larger) data by multiple and refined (e.g. goeBURST) phylogenetic tools, which provided better linkage among STs. Recently, association of Indian *B*. *pseudomallei* isolates with Southeast Asian isolates have been reported by others as well [20].

MLST has been widely applied to determine the clonal relationship among strains of *B*. *pseudomallei* and other various clinically relevant bacterial species. The online MLST databases facilitated the sharing and analysis of MLST data. However, MLST is based on the sequencing of only seven (housekeeping) genes, therefore, has low resolution. MLST has added limitations in phylogeographic analysis of *B*. *pseudomallei*, because this bacterium has high rates of recombination and instances of homoplasy. Therefore, it is possible that the isolates may share the same ST despite being distinct genetically. Many Indian STs including a few predominant Indian STs like ST1368 and ST1373 were found to cluster with Australian isolates. Grouping of Asian isolates with isolates of Australian origin by eBURST analysis has previously been shown and two STs shared between Australia and Cambodia were found to be genetically unrelated on the whole-genome level [23]. Whole genome sequencing (WGS) based SNP typing provides higher resolution and can be more robust and accurate to identify the origin of strains. WGS based methods are being used to study the phylogeny and evolution of bacteria in the newer studies [24,25]. It would be interesting to find out in future how WGS can be used to elucidate the clustering of Indian isolates with Asian (or Australian) isolates and also to find out (or rule out) the clustering of those Indian STs that overlapped/grouped with Australia.

### Conclusions

Overall, results of this study suggest that Indian *B*. *pseudomallei* isolates are closely related with lesser heterogeneity among them and belong to single group. However, it appears that there are a few outliers which are distantly related to the majority of Indian STs. The results further suggest that Indian isolates are closely related to *B*. *pseudomallei* isolates from Southeast Asia, particularly from South Asia, and there seems to be migration of bacteria between India and other Southeast Asian countries. However, due to low resolution of MLST, future studies should focus on WGS based SNP typing to find out (or rule out) the clustering of Indian *B*. *pseudomallei* with Asian or Australian isolates.

## Supporting information

S1 Table Isolates study based on the clinical condition, geographical location, year of isolation, YLF/BTFC genotype and sequence type. (DOCX)

S2 Table Description of STs based on eBURST analysis. (DOCX)
