# Supplementary material for: Molecular analysis of clinical Burkholderia pseudomallei isolates from southwestern coastal region of India, using multi-locus sequence typing
Source: PLoS Negl Trop Dis. 2018 Nov 12;12(11):e0006915. doi: 10.1371/journal.pntd.0006915 (PMC6258418; doi:10.1371/journal.pntd.0006915)
Supplement: S1 Table — (DOCX) [file pntd.0006915.s001.docx]

**Table S1:** Isolates study based on the clinical condition, geographical location, year of isolation, YLF/BTFC genotype and sequence type

| **Strain name** | **Isolate ID** | **Type of infection** | **Specimen** | **Geographical location place, State** | **Year of isolation** | **YLF/**  **BTFC** | **ST** |
| --- | --- | --- | --- | --- | --- | --- | --- |
| 92 | 5496 | Localized | Pus | Udupi, Karnataka | 2010 | YLF | 1368 |
| 108 | 5497 | Localized | Pus | Ankola, Karnataka | 2012 | YLF | 1507 |
| 112 | 5498 | Systemic | Blood | Kumta, Karnataka | 2012 | YLF | 1375 |
| 114 | 5499 | Localized | Pus | Kerala | 2012 | YLF | 1508 |
| 115 | 5500 | Systemic | Blood | Kumta, Karnataka | 2012 | YLF | 1507 |
| 121 | 5501 | Systemic | Blood | Goa | 2012 | YLF | 1506 |
| 122 | 5502 | Localized | Pus | Udupi, Karnataka | 2012 | YLF | 960 |
| 124 | 5496 | Pulmonary | Sputum | Kumta, Karnataka | 2012 | YLF | 1141 |
| Ma6 | 5503 | Localized | Pus | Goa | 2008 | YLF | 550 |
| Ma7 | 5504 | Systemic | Blood | Shimoga, Karnataka | 2008 | YLF | 1368 |
| Ma11 | 5505 | Systemic | Blood | Shimoga, Karnataka | 2009 | YLF | 293 |
| Ma12 | 5506 | Systemic | Blood | Kerala | 2009 | YLF | 405 |
| Ma13 | 5507 | Systemic | Blood | Shimoga, Karnataka | 2009 | YLF | 293 |
| Ma14 | 5508 | Localized | Pus | Bhatkal, Karnataka | 2008 | YLF | 1368 |
| Ma22 | 5509 | Pulmonary | Sputum | Koppal, Karnataka | 2010 | YLF | 405 |
| Ma25 | 4178 | Systemic | Blood | Chickmagalur, Karnataka | 2010 | YLF | 1373 |
| Ma26 | 4179 | Localized | Pus | Shimoga, Karnataka | 2010 | YLF | 1374 |
| Ma27 | 4180 | Localized | Pus | Shimoga, Karnataka | 2010 | YLF | 1368 |
| Ma28 | 4181 | Systemic | Blood | Kundapura, Karnataka | 2010 | YLF | 1375 |
| Ma30 | 4182 | Localized | Pus | Puducherry | 2011 | YLF | 1368 |
| Ma31 | 4790 | Systemic | Blood | Chickmagalur, Karnataka | 2011 | YLF | 1509 |
| Ma32 | 5510 | Localized | Pus | Kumta, Karnataka | 2011 | YLF | 1507 |
| Ma33 | 4791 | Localized | Pus | Honnavar, Karnataka | 2011 | YLF | 1510 |
| Ma34 | 5511 | Systemic | Blood | Kerala | 2011 | YLF | 859 |
| Ma35 | 5512 | Systemic | Blood | Karwar, Karnataka | 2012 | YLF | 1051 |
| Ma36 | 5513 | Systemic | Blood | Ankola, Karnataka | 2012 | YLF | 1372 |
| Ma37 | 4792 | Systemic | Blood | Sirsi, Karnataka | 2012 | YLF | 1511 |
| Ma38 | 4793 | Localized | Pus | Shimoga, Karnataka | 2012 | YLF | 1512 |
| Ma39 | 5514 | Systemic | Blood | Udupi, Karnataka | 2012 | YLF | 1372 |
| Ma40 | 5515 | Localized | Pus | Kumta, Karnataka | 2012 | YLF | 1512 |
| Ma41 | 5516 | Localized | Pus | Shimoga, Karnataka | 2012 | YLF | 1370 |
| Ma42 | 5517 | Systemic | Blood | Kerala | 2012 | YLF | 468 |
| Ma43 | 5518 | Systemic | Blood | Kumta, Karnataka | 2012 | YLF | 1375 |
| Ma44 | 4794 | Systemic | Blood | Udupi, Karnataka | 2012 | YLF | 1513 |
| Ma45 | Ma-45 | Systemic | Blood | Shimoga, Karnataka | 2012 | YLF | 1512 |
| Ma46 | 4795 | Pulmonary | Sputum | Goa | 2012 | YLF | 1514 |
| Ma47 | 5520 | Localized | Pus | Karwar, Karnataka | 2012 | YLF | 42 |
| Ma48 | 4796 | Pulmonary | Sputum | Kumta, Karnataka | 2012 | YLF | 1515 |
| Ma49 | 4797 | Localized | Pus | Udayavara, Karnataka | 2012 | YLF | 1516 |
| Ma51 | 5521 | Systemic | Blood | Kota, Rajasthan | 2012 | YLF | 1368 |
| Ma52 | 5522 | Localized | Pus | Goa | 2013 | YLF | 1513 |
| Ma53 | 5523 | Localized | Pus | Udupi, Karnataka | 2013 | YLF | 1368 |
| Ma54 | 4798 | Systemic | Blood | Udupi, Karnataka | 2013 | YLF | 1517 |
| Ma55 | 5524 | Localized | Pus | Udupi, Karnataka | 2013 | YLF | 1368 |
| Ma56 | 5525 | Localized | Pus | Udupi, Karnataka | 2013 | YLF | 1368 |
| Ma57 | 5526 | Systemic | Blood | Udupi, Karnataka | 2013 | YLF | 1368 |
| Ma58 | 5527 | Localized | Pus | Parkala, Karnataka | 2013 | YLF | 42 |
| Ma59 | 5528 | Localized | Pus | Kundapura, Karnataka | 2013 | YLF | 1368 |
| Ma60 | 5529 | Systemic | Blood | Manur, Karnataka | 2013 | YLF | 1517 |
| Ma61 | 4799 | Systemic | Blood | Bhatkal, Karnataka | 2013 | YLF | 1518 |
| Ma62 | 5530 | Systemic | Blood | Udupi, Karnataka | 2013 | YLF | 42 |
| Ma63 | 4800 | Systemic | Blood | Udupi, Karnataka | 2013 | YLF | 1519 |
| Ma64 | 5531 | Localized | Pus | Davengere, Karnataka | 2013 | YLF | 1373 |
| Ma65 | 4801 | Systemic | Blood | Kundrapur, Karnataka | 2013 | YLF | 1520 |
| Ma66 | 5532 | Localized | Pus | Kumta, Karnataka | 2013 | YLF | 1373 |
| Ma67 | 5533 | Localized | Pus | Udupi, Karnataka | 2013 | YLF | 1373 |
| Ma68 | 5534 | Systemic | Blood | Davengere, Karnataka | 2013 | YLF | 1478 |
| Ma69 | 5535 | Systemic | Blood | Udupi, Karnataka | 2013 | YLF | 1478 |
| Ma70 | 5536 | Systemic | Blood | Udupi, Karnataka | 2013 | YLF | 1478 |
| Ma71 | 5537 | Systemic | Blood | Udupi, Karnataka | 2014 | YLF | 1478 |
| Ma72 | 5538 | Pulmonary | Sputum | Honnavar, Karnataka | 2014 | YLF | 42 |
| Ma73 | 5539 | Localized | Pus | Udupi, Karnataka | 2014 | YLF | 124 |
| Ma74 | 5540 | Pulmonary | Sputum | Calicut, Karnataka | 2014 | YLF | 124 |
| Ma75 | 5541 | Localized | Pus | Shimoga, Karnataka | 2014 | YLF | 124 |
