# Supplementary material for: Molecular analysis of clinical Burkholderia pseudomallei isolates from southwestern coastal region of India, using multi-locus sequence typing
Source: PLoS Negl Trop Dis. 2018 Nov 12;12(11):e0006915. doi: 10.1371/journal.pntd.0006915 (PMC6258418; doi:10.1371/journal.pntd.0006915)
Supplement: S2 Table — (DOCX) [file pntd.0006915.s002.docx]

**Table S2:** Description of STs based on eBURST analysis

| ST | Frequency | SLV | DLV | TLV | Satellite |
| --- | --- | --- | --- | --- | --- |
| 1368 | 11 | 7 | 4 | 7 | 9 |
| 124 | 3 | 4 | 4 | 6 | 13 |
| 42 | 4 | 4 | 2 | 9 | 12 |
| 293 | 2 | 3 | 10 | 7 | 7 |
| 1513 | 2 | 3 | 8 | 6 | 10 |
| 550 | 1 | 3 | 7 | 14 | 3 |
| 1373 | 4 | 3 | 6 | 7 | 11 |
| 1518 | 1 | 3 | 5 | 5 | 14 |
| 1512 | 3 | 3 | 4 | 8 | 12 |
| 1511 | 1 | 3 | 1 | 4 | 19 |
| 1517 | 2 | 2 | 9 | 9 | 7 |
| 1507 | 3 | 2 | 9 | 5 | 11 |
| 1372 | 2 | 2 | 7 | 5 | 13 |
| 1510 | 1 | 2 | 6 | 8 | 11 |
| 405 | 2 | 2 | 6 | 7 | 12 |
| 1515 | 1 | 2 | 6 | 6 | 13 |
| 1508 | 1 | 2 | 5 | 7 | 13 |
| 1520 | 1 | 2 | 3 | 7 | 15 |
| 1519 | 1 | 2 | 2 | 12 | 11 |
| 1051 | 1 | 2 | 2 | 7 | 16 |
| 1516 | 1 | 1 | 9 | 7 | 10 |
| 1370 | 1 | 1 | 8 | 13 | 5 |
| 1514 | 1 | 1 | 6 | 7 | 13 |
| 960 | 1 | 1 | 5 | 8 | 13 |
| 1509 | 1 | 1 | 4 | 6 | 16 |
| 468 | 1 | 1 | 3 | 9 | 14 |
| 1375 | 3 | 1 | 2 | 3 | 21 |
| 1478 | 4 | 1 | 1 | 7 | 18 |
| 1141* | - |  |  |  |  |
| 1374* | - |  |  |  |  |
| 1506* | - |  |  |  |  |
| 859* | - |  |  |  |  |

*Singleton STs
